# The Effect of Perioperative Auditory Stimulation with Music on Procedural Pain: A Narrative Review

**DOI:** 10.1007/s11916-023-01138-x

**Published:** 2023-07-06

**Authors:** Fabienne C. S. Frickmann, Richard D. Urman, Kaya Siercks, Gabriel Burgermeister, Markus M. Luedi, Friedrich E. Lersch

**Affiliations:** 1 Department of Anaesthesiology and Pain Medicine, Inselspital, Bern University Hospital, University of Bern, Freiburgstrasse, Bern, 3010 Switzerland; 2 Department of Anesthesiology, The Ohio State University, Columbus, OH 43210 USA; 3 Department of Anaesthesiology and Pain Medicine, Cantonal Hospital of St. Gallen, St. Gallen, 9007 Switzerland

**Keywords:** Music, Acute pain, Pain pathways, Perioperative care, Analgesia, Active inference, Nociception

## Abstract

**Purpose of Review:**

Music therapy has seen increasing applications in various medical fields over the last decades. In the vast range of possibilities through which music can relieve suffering, there is a risk that—given its efficacy—the physiological underpinnings are too little understood. This review provides evidence-based neurobiological concepts for the use of music in perioperative pain management.

**Recent Findings:**

The current neuroscientific literature shows a significant convergence of the pain matrix and neuronal networks of pleasure triggered by music. These functions seem to antagonize each other and can thus be brought to fruition in pain therapy. The encouraging results of fMRI and EEG studies still await full translation of this top-down modulating mechanism into broad clinical practice.

**Summary:**

We embed the current clinical literature in a neurobiological framework. This involves touching on Bayesian “predictive coding” pain theories in broad strokes and outlining functional units in the nociception and pain matrix. These will help to understand clinical findings in the literature summarized in the second part of the review. There are opportunities for perioperative practitioners, including anesthesiologists treating acute pain and anxiety in emergency and perioperative situations, where music could help bring relieve to patients.

## Introduction

Pain is a fundamentally subjective experience. This is reflected in the revised International Association for the Study of Pain (IASP) definition of pain [1], where physiological processes of nociception are distinguished from the sensation of pain as an emotional experience. The latter distillates multiple pain mechanisms [2, 3] and converging pain pathways [4–6], which individually or collectively influence pain perception with varying degrees of importance. Thus, the embodiment of pain is comprised of different aspects: affective, perceptive, attentive, discriminative, and vegetative components [7•]. The multidimensional nature of pain facilitates external modulatory access to pain processing. This has led to an increased interest in and growing body of literature on non-pharmacological pain-modulating interventions [8–10]. We illustrate neurobiological concepts, clinical context, and possible practical applications of the pain-modulating effects of music, focusing on the intra- and postoperative setting [11, 12•, 13•]. These are outlines from the viewpoint of anesthesiologists administering music as part of a multimodal anesthesia and pain analgesia approach [14, 15]. We start from current pain theories embedded in the framework of predictive coding models for pain theory [16•]. A discussion of neurobiological modulation of pain perception and sensation through music [17•, 18, 19] and its measurability through electroencephalography (EEG) [20•] and vegetative symptoms follows. We provide an overview on clinical trials, using music as a remedy or a complimentary treatment for acute pain [21–26]. The evidence presented is meant to inform practicing clinicians adopting music into their non-pharmacologic therapeutic approach to manage pain. We will stress the application of music to the analgesic armamentarium. Its effectiveness treats pain while respecting the patient’s agency [27•].

## The Neurobiology of Pain

### The Pain Matrix

**Fig. 1:**
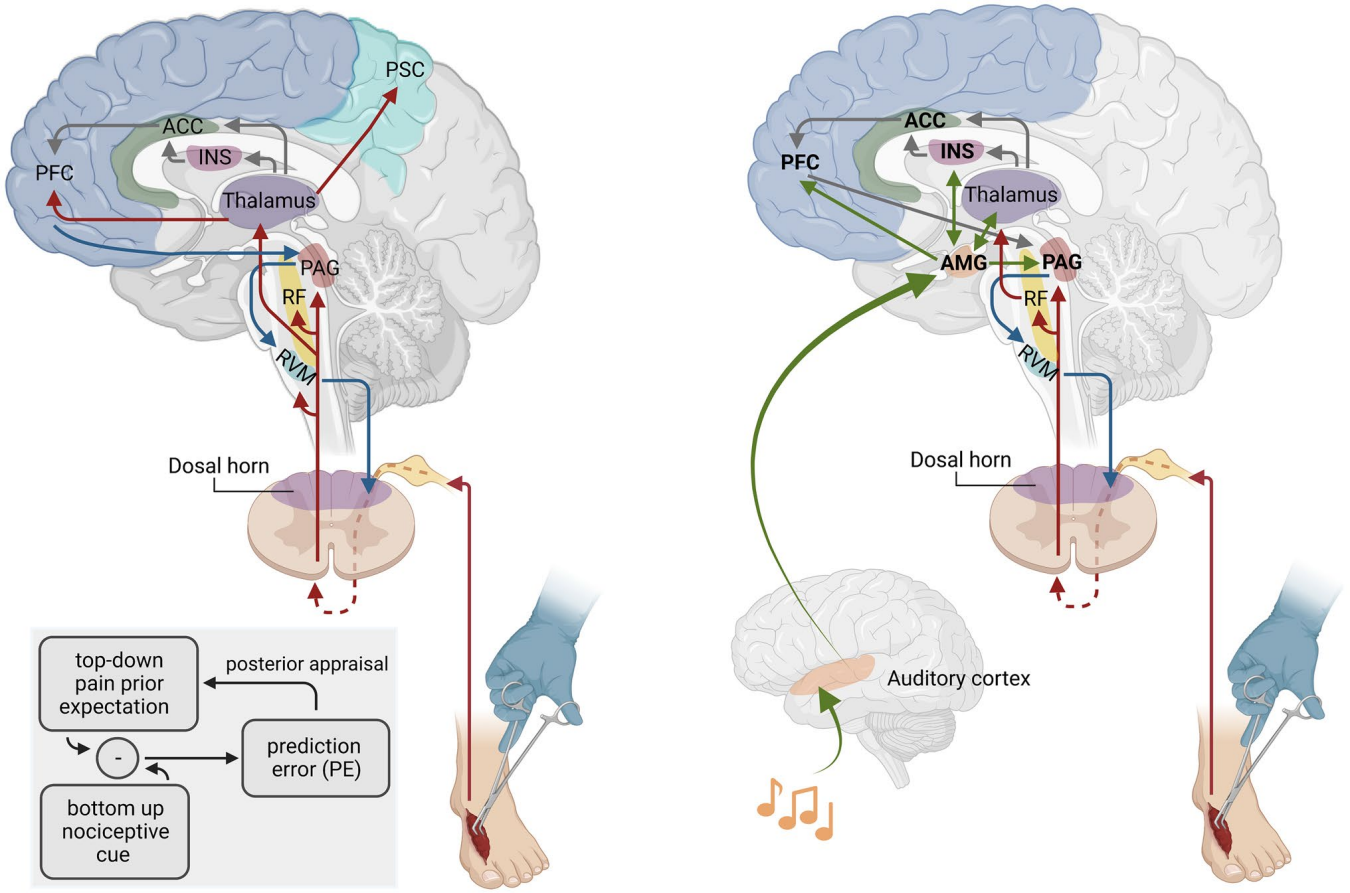
The pain matrix (left) and musical pain modulation (right). Sensory informations about a noxious stimulus travel on ascending pathways (red arrows) via relay stations (RF = reticular formation, PAG = periaqueductal grey, RVM = rostral ventromedial medulla, INS = insular cortex, ACC = anterior cingulate cortex, PFC = prefrontal cortex, PSC = primary somatosensory cortex). The assessment of the stimulus is modulated through subcortical and cortical connections (grey arrows) and regulatory descending pathways (blue arrows). Musical sounds stimulate the auditory cortex, which has connections to the amygdala (AMG). AMG interconnections are indicated with green arrows. Top-down reactions to a simultaneously occurring noxious stimulus are modulated through emotional and attentional changes caused by the musical sounds. The prediction model for pain perception (bottom left). Created with BioRender.com

**Fig. 2:**
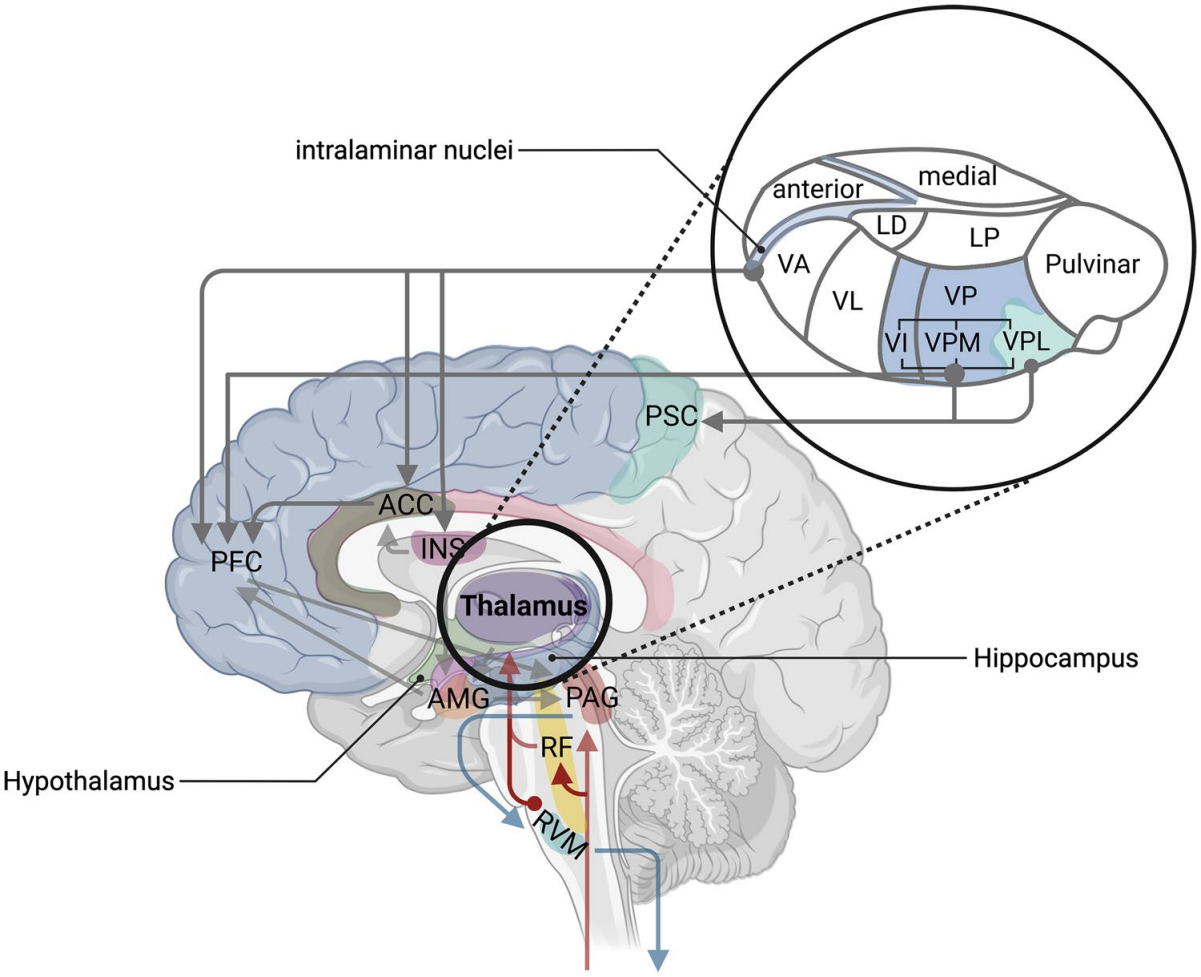
Detailed schematic representation of thalamic nuclei and structures of the limbic system involved in the pain processes (anterior = anterior nucleus, medial = medial nucleus, LD = laterodorsal nucleus, LP = lateral posterior nucleus, ventral anterior nucleus, VA = ventral anterior nucleus, VL = ventral lateral nucleus, VP = ventral posterior complex, VI = ventral intermediate nucleus, VPM = ventral posteromedial nucleus, VPL = ventral posterolateral nucleus). Connections to subcortical and cortical pain regions marked with grey arrows (RF = reticular formation, PAG = periaqueductal grey, RVM = rostral ventromedial medulla, INS = insular cortex, ACC = anterior cingulate cortex, PFC = prefrontal cortex, PSC = primary somatosensory cortex, AMG = amygdala). Created with BioRender.com

Following a noxious stimulus, information on the tissue injury is transmitted from the periphery to the posterior horn of the spinal cord, where primary pain nerve fibers end and synapse. The secondary neuron crosses over and ascends contralaterally in the ascending spinal pathways of the anterolateral system. Here, the signal can travel one of three ways:

1. The primary nociceptive path of this ascending system is the spinothalamic tract. Information on this path travels either towards reticular formation (RF) to modulate arousal, the periaqueductal gray (PAG) in the mesencephalon (also involved in the autonomic nervous system and threat response) [28], or is further carried to the ventroposterolateral (VPL) nucleus, medial nucleus of the posterior complex (POm), and central lateral (CL) (intralaminar) nuclei of the thalamus [29]. From here, information can be disseminated to the primary somatosensory cortex (PSC, discriminative information on noxious stimulus, i.e., precise location) and to subcortical and cortical areas, including the prefrontal cortex (PFC), hypothalamus, and hippocampus (Fig. 1). This allows for generation of an emotional reaction to the noxious stimulus, modulation, and finally pain perception and vegetative responses [4, 5, 30]. The intralaminar nuclei of the thalamus have connections to the insular cortex and cingulate cortex (Fig. 2). These areas deal with information on the quality of the pain (dull, burning, crude touch) and are responsible for emotional associations.
2. Spinal trigeminal projections travel to the RF and continue with other afferents, ending close to the PAG or in the ventroposteromedial nucleus (VPM; information on sharp pain) and intralaminar nuclei of the thalamus. The VPM also has connections to the primary somatosensory cortex.
3. The spinoreticular tract carries information to nuclei in the reticular formation and rostral ventromedial medulla (RVM) and from there to the intralaminar nuclei of the thalamus. These pathways create a multisynaptic circuit, the *pain matrix* [31]. Signal transmission in the ascending pain pathways is facilitated by norepinephrine as a neurotransmitter [29].

### Top-Down Control

Descending pathways originating segmentally in the spinal cord [32], as well as from the mesencephalon, specifically the periaqueductal gray (PAG) [33–35] and medulla oblongata (rostroventral medulla, RVM), the hypothalamus, and the prefrontal cortex, allow for further modulation of pain perception and sensation [36]. This second part of the pain pathway creates a top-down control [36–38] and grants access points for external pain modulation. Multiple receptor types and neurotransmitters are involved in these modulatory processes, including γ-aminobutyric acid (GABA), facilitating interaction between descending fibers and afferent pathways in the dorsal horn, blocking or amplifying pain signals. These are then further transmitted to the PAG and amygdala among others, for re-assessment [29]. Figure 1 displays the pain pathways and matrix.

## Predictive Coding Models for Pain Theory

When Gardner et al. published their study on auditory analgesia in dentistry in 1959 [39], a vivid debate ensued about therapeutic potential and the physiology of analgesia mediated by music and sound [40]. The initial study had shown significant analgesia in more than 5000 patients undergoing dentistry, while listening to waterfall noise with underlying relaxing music. The patients were modulating the intensity of the noise overlayed on soothing music. It was argued that attention and concentration afforded to this task dissociated patients from nociceptive cues. Patients reported that pain was present but of inconsequential character. Another group of dentists proposed that auditory analgesia could modulate pain perception by (1) cross-modality masking, by (2) distraction or dissociation, or (3) by suggestion of positive emotions. All three could alter the threshold for pain perception. These questions remain unanswered but resonate with new concepts of pain perception and musical emotions in a very interesting way. Whether music should block out nociceptive sensations, alter the weighting of stimuli (gain) or just alter the interpretation of a potentially painful stimulus remains unclear. Current theories of predictive coding, Bayesian inference in the brain, and reinforcement learning offer conceptual depth in reflecting on the problem both in clinical-practical and scientific terms. They offer a different view on pain genesis, assisting with its comprehension but not intending to deny biomedical concepts. Under a “Bayes’ brain” assumption [41] to “make sense of the world”, the brain must integrate all sensory inputs with (social) setting information and preexisting knowledge [42•]. Adapted from the inference statistical theory, the brain processes pain by estimating the probability that a perception (unpleasant stimulus) is true, based on preexisting conceptions and knowledge [43]. This Bayesian analysis is applied continuously to incoming data (sensory inputs), generating the top-down signaling cascade. These correspond to the brain’s “predictions” about the reality. When ascending information from the afferent pain pathways clashes with the brain’s predictions—as pain perception—about an incoming stimulus, a so called “prediction error” occurs, prompting re-analysis with new top-down signals and closing the loop of the multisynaptic pain circuitry (Fig. 1) [44•].

## Neurobiological Modulation of Pain Perception and Sensation Through Music

Music as part of a multimodal intra- and postoperative pain management concept has significant effects on both subjective pain levels and analgesia requirements postoperatively [45]. The modulatory influences of music on the brain seem to be multidimensional [46], encompassing effects on mood and arousal [47] and focus of attention [46].

Pain perception is dependent on the emotional valence (positive, negative, neutral) of a stimulus [48]. By changing the emotional significance of a painful stimulus, through distraction, induction of a positive frame of mind and reduced responsiveness, music can modify pain perception [18, 20•]. Comparing the neuronal networks involved in pain appraisal with those of music evaluation in the brain shows significant convergence. Especially the orbitofrontal and insular cortex, the cingulate gyrus, the amygdala, and the periaqueductal gray seem to determine both the affective valence of a nociceptive cue and pleasant music [49•]. Activity and connectivity patterns of these neuronal hubs when concomitantly activated by nociception and pleasant music may determine the threshold of a perception to be felt as pain. This corresponds to “precision” in predictive coding theory. EEG findings described by Lu et al. suggest that listening to self-chosen music directly before a painful stimulus is associated with a decrease in alphaoscillations corresponding to an activation of brain areas related to music processing, mainly the prefrontal cortex and anterior cingulate cortex [20•]. Thus, it can be hypothesized that music activates brain regions involved in descending pain pathways and amplifies top-down pain modulation.

The provoked change in emotional appraisal of an unpleasant stimulus can be viewed in the context of the positive valence musical sounds can have on the state of emotional arousal. Musical sounds perceived as pleasant by an individual can trigger a dopamine-fueled reward cascade. It leads to positive emotions and so-called musical chills as the highest state of pleasure caused by music [50–52] and consequently causes a shift in baseline mood and arousal. This phenomenon was electroencephalographically measured by Chabin et al. [50]. The authors were able to demonstrate a distinct neuronal activity represented by an increase in theta-bands in the prefrontal cortex amongst others, during subjective positive emotional arousal, and linked this specific EEG pattern to a state of “pleasure” [50]. Neurobiologically, connectivity between the auditory cortex, the amygdala, and a plethora of other regions involved in the emotional processing allows for modulation of stimulus perception. Consequently, the cortical representation of a painful stimulus and thus its significance and evaluation at any given time is related to the emotional state of the individual [18]. Music induces “pleasurable chills”, and this positive emotional arousal state could be identified by the distinct EEG pattern, and used to modulate pain induced neuronal activity. However, it is important to consider that both musical experiences and pain are ultimately subjective. Objectifying both is thwarted by the difficulties of quantifying the emotional dimensions an organism goes through while suffering pain or enjoying music. Along with dopamine-reward circuits, effects of music on other neurotransmitter and neuroendocrine systems have been proposed. Nilsson et al. measured rising oxytocin levels during relaxation with calming music at 60 to 80 bpm and 50 to 60 dB [53]. In addition, a stimulation of endogenous opioid release [54] has been theorized as a link to top-down pain-regulating pathways, but a direct connection between incoming auditory stimuli and decrease in pain response and perception is yet to be shown [55•].

The second pain-modulating component of listening to music presents as a shift in attentional focus (distraction). In neuroimaging methods (magnetoencephalography, MEG), this seems to be characterized by a decrease in pain-induced delta-amplitudes in the cingulate gyrus and the insular cortex. This shifts attention away from pain towards music [46].

Present-day experimental findings speak for more complicated neuropsychological processes involving preexisting expectations responsible for the analgesic effect of pleasant sounds [55•]. This is supported by the predictive model of pain perception, viewing the brain not as a behavioralist black box [56] where certain inputs mechanistically trigger an outcome, but rather as a complex matrix of prior experiences and assumptions, leading to a co-analgesic effect [42•, 44•]. The neurobiological base to this psychological theory may be viewed to be the interconnections of the pain network, the relay of spino-thalamic-cortical and subcortical connections [42•]

## Measuring Pain Modulation: Neuroimaging, EEG, Hormonal, and Vegetative Markers

Functional magnetic resonance imaging (fMRI) analysis has shown different activation patterns of spinal and cortical areas to painful stimuli with music compared to without music [57]. This can be seen as neurobiological evidence for the hypothesis that music changes (secondary) pain processing. However, neuroimaging for pain has its limitations [58] as shown in numerous, contradictory, studies over the past years trying to isolate brain regions selective for pain. These limitations mainly arise from “reverse inference” used to deduce the meaning of certain signals in fMRI. There seems to be debate amongst neuroscientific researchers as to whether MRI signals can be conclusively assigned to specific neurocellular processes [59, 60].

EEG signals obtained during and after a painful stimulus furthermore suggest that preferred music decreases the unpleasantness (subcortical *affective* pain component of the amygdala, insular cortex, and PAG), though not the perceived pain intensity (cortical somatosensory discriminatory and *attentional* prefrontal pain component) [20•, 48, 61]. Lu et al. [20•] could not conclude whether an alteration of the emotional state of the participants or a diversion of attention away from the painful stimulus evoked a decrease in pain sensation. More recent findings lean towards a mechanism where assigned emotion influences the affective component of pain (unpleasantness) more than the attentional component [48]. Williams and Hine [26] found that across 39 articles, distraction (in terms of gate control theory [36]) was mostly used as an underlying mechanism to explain the analgetic effectiveness of music, followed by relaxation, emotional shift, oscillatory entrainment of neuronal activity, and endogenous analgesics stimulated by music (opioid receptor expression, interleukine-6, and morphine-6 glucuronide [62] or increase in endorphins [63, 64]). The analysis showed that despite the effort of explaining the underlying mechanisms, most of the studies did not select music with parameters conducive to these processes [26]. For example, if entrainment is to be considered a valid contributor to the pain-relieving effect of intraoperative music, this connection should be explored further through EEG studies under sedation and general anesthesia, respectively. It is hypothesized that music, with a steady rhythm and frequencies around 60 to 80 bpm, could be ideal to promote a decrease in sympathetic activity and thus stress [26, 62, 65, 66].

Brain regions involved in pain processing, namely, the PFC, amygdala, and hippocampus, are also involved in stress response [67]. These in turn have close connections to the hypothalamus and thus to the hypothalamic–pituitary–adrenal (HPA) axis and the sympathetic nervous system (SNS) by signaling to the adrenal glands [68]. When matched for time of day and circadian rhythms, Koelsch et al. [69] found lower intraoperative cortisol levels in patients receiving music during general anesthesia as well as deeper sedation stages as measured by bispectral indices (BIS). This suggests lower sedative requirements and consequently a lower activation of the SNS in patients undergoing musical intervention. This supports earlier cortisol level results from Nilsson et al. [70], but contradicts others showing no effect on stress hormone levels and BIS [71].

Measuring cortical electrical signals has been shown to be a useful and accessible tool to determine effects of both musical [72] and painful stimuli on brain activity. We propose that future studies use raw EEG sedation stages and wave patterns as an additional feasible outcome measure for intraoperative stress and pain perception. Co-analgesic effects of music characteristics matching specific EEG patterns could be analyzed for their potential to reduce pain unpleasantness.

## Clinical Context: Opioid-Sparing Effects and Postoperative Recovery

To objectify the clinical analgesic effect of perioperative music interventions, the following outcome parameters seem to have been most useful: the effect on postoperative pain during the first 24 h after general anesthesia (self-reported on the NRS, quantified by reduction in opioid requirements [13•]), the effect on intraoperative analgesic requirements under general anesthesia as quantified by intraoperative opioid dosage, the influence on the duration of the hospital stay, and the effect on successful early mobilization. The most feasible measure of the effect on intraoperative stress and pain reaction to surgical intervention seems to be heart rate variability [73], with neuroendocrine measurements not being easily applicable to the daily anesthesiology practice.

So far, no significant reduction in length of hospital stays, time spent in the intensive care unit, or overall costs during hospitalization through music-analgesia could be demonstrated [13•, 74].

Most of the systematic reviews and meta-analyses, including recent publications by Fu et al. [13•] and Dale [86•], found a reduction in postoperative opioid requirements after perioperative music across all analyzed studies [75], which cemented the conclusions of Kühlmann et al. [9] pointing towards significant reductions in pain levels in patients receiving musical interventions during general anesthesia. This contrasts with an earlier meta-analysis which did not find significant differences in pain levels for patients being played music during colonoscopies [76]. However, the very limited target group must be considered here. A subsequent analysis of pain reduction through music during different endoscopic procedures showed that no significant analgesic-saving effect could be detected, especially during colonoscopies and bronchoscopies [77]. Further research is needed to determine patient subgroups who would most benefit from peri-interventional and perioperative music. Currently available publications of clinical trials and randomized-controlled studies in adults are predominantly published in the field of orthopedic surgery, urology and obstetrics, and ophthalmology, as well as interventional radiology and heart and thoracic surgery [78–84]. Orthopedic and thoracic surgery focused on postoperative musical interventions during recovery [10], although all studies found reductions in procedural pain through music.

## Clinical Applications

The currently available body of literature is very heterogeneous, which makes concrete conclusions for practice only possible to a limited extent. In order to make use of music as a tangible co-analgesic, a feasible framework for such intervention needs to be defined. Further research needs to determine the differences and optimal timing of the intervention (solely preoperatively, solely intraoperatively, solely postoperatively, from induction of general anesthesia or sedation until after emergence). Compelling evidence for preserved auditory sensory perception under general anesthesia [85] suggests that intraoperative acoustic stimulation with music could maintain its pain-modulating effect even during deep sedation [74]. Comprehensively, the timing of the musical intervention seems to have only marginal influence on postoperative pain reduction [74]. Additionally, it needs to be determined which type of music or sounds proves to be the most beneficial (patient’s choice, classical or electronic sounds of a specific frequency, chosen from preselected playlist or free choice, nature sounds, white noise) and for which intervention [26]. It must be noted that nature sounds have not consistently shown the same analgesic effect as self-selected music [55•]. Fu et al. in their meta-analysis excluded all studies that used nature sounds during intervention and focused solely on recorded music [13•]. Future research should consider the complex mechanisms of music to determine whether the melody, harmony, or rhythm might be the determining factor for its analgesic effect [26, 75]. Overall, the evidence points to patient-preferred or self-selected music, respectively, as being the most effective in reducing postoperative pain [9, 20•, 86•].

Across all systematic reviews and meta-analysis currently available, no adverse effects of musical interventions have been reported.

There are currently two ongoing studies registered with ClinicalTrials.gov on perioperative musical intervention during general anesthesia [87, 88].

## Summary and Outlook

There appears to be some consensus that auditory stimulation with music during or after surgical interventions, under sedation or general anesthesia, can be considered an effective and low-cost, low-side-effect complimentary treatment to alleviate procedural and postoperative pain. Musical interventions have a low-threshold applicability in everyday clinical practice. Offering music via headphones during operations under regional anesthesia, with or without sedation, is already common practice. Anesthesia providers seem to hope for a relaxing and distracting effect, especially during operations with high noise levels, such as orthopedics. Patients can play their own music via their mobile phones or choose from a provided music library with a selection of genres and artists. In our experience, clinicians do not seem to have concrete expectations of reduced intraoperative opioid or sedative requirements when offering musical stimulation to their patients in the operating room. Our aim was to concretize anecdotal assumptions of musical analgesia, encourage anesthesia teams to systematically use music as a co-analgesic, and suggest ways in which future research in this area can unite neuropsychological and biological, philosophical, and anesthesiologic theories of pain and consciousness and further establish pain modulatory approaches.

## Data Availability

Not applicable.
